# Sparsity estimation from compressive projections via sparse random matrices

**DOI:** 10.1186/s13634-018-0578-0

**Published:** 2018-09-10

**Authors:** Chiara Ravazzi, Sophie Fosson, Tiziano Bianchi, Enrico Magli

**Affiliations:** 1 National Research Council of Italy, IEIIT-CNR, c/o Politecnico di Torino, Corso Duca degli Abruzzi 24, Torino, 10129 Italy; 2 Politecnico di Torino, DAUIN, Corso Duca degli Abruzzi 24, Torino, 10129 Italy; 3 Politecnico di Torino, DET, Corso Duca degli Abruzzi 24, Torino, 10129 Italy

**Keywords:** Sparsity recovery, Compressed sensing, High-dimensional statistical inference, Gaussian mixture models, Maximum likelihood, Sparse random matrices

## Abstract

The aim of this paper is to develop strategies to estimate the sparsity degree of a signal from compressive projections, without the burden of recovery. We consider both the noise-free and the noisy settings, and we show how to extend the proposed framework to the case of non-exactly sparse signals. The proposed method employs *γ*-sparsified random matrices and is based on a maximum likelihood (ML) approach, exploiting the property that the acquired measurements are distributed according to a mixture model whose parameters depend on the signal sparsity. In the presence of noise, given the complexity of ML estimation, the probability model is approximated with a two-component Gaussian mixture (2-GMM), which can be easily learned via expectation-maximization.

Besides the design of the method, this paper makes two novel contributions. First, in the absence of noise, sufficient conditions on the number of measurements are provided for almost sure exact estimation in different regimes of behavior, defined by the scaling of the measurements sparsity *γ* and the signal sparsity. In the presence of noise, our second contribution is to prove that the 2-GMM approximation is accurate in the large system limit for a proper choice of *γ* parameter. Simulations validate our predictions and show that the proposed algorithms outperform the state-of-the-art methods for sparsity estimation. Finally, the estimation strategy is applied to non-exactly sparse signals. The results are very encouraging, suggesting further extension to more general frameworks.

## Introduction

Compressed sensing (CS) [1, 2] is a novel signal acquisition technique that recovers an unknown signal from a small set of linear measurements. According to CS, if a signal having dimension *n* is known to be sparse, i.e., it has only *k*≪*n* non-zero entries when represented by a suitable basis, then it can be efficiently recovered using only *m*≪*n* linear combinations of the signal entries, provided that these linear projections are sufficiently incoherent with respect to the signal basis.

In most of CS applications, it is usually assumed that an upper bound on the sparsity degree *k* is known before acquiring the signal. However, some signals may have a time-varying sparsity, as in spectrum sensing [3], or spatially varying sparsity, as in the case of block-based image acquisition [4]. Since the number of linear measurements required for the recovery depends on the sparsity degree of the signal [5], the knowledge of *k* is crucial to fully exploit the potential of CS.

In many recovery algorithms, the optimal tuning of parameters requires the knowledge of the degree of sparsity of the signal. For example, in Lasso techniques [6], a parameter *λ* related to *k* has to be chosen [7], whereas for greedy algorithms, such as orthogonal matching pursuit (OMP) [8] or compressive sampling matching pursuit (CoSaMP) [9], the performance and the number of iterations depend on *k*.

The ability to estimate the signal sparsity degree directly from a small number of linear measurements can represent an important tool in several promising applications. One of the most obvious applications is the possibility to dynamically adapt the number of measurements acquired by a CS instrument, e.g., an imager, to the estimated signal sparsity. We can envisage a system that acquires linear measurements in a sequential way and continuously updates the estimated sparsity according to the new measurements. The acquisition can stop as soon as the number of acquired measurements is enough to guarantee the correct reconstruction of a signal based on the estimated sparsity.

Other applications may include the possibility of comparing the support of two sparse signals from their measurements. Due to the linearity of the sparse signal model, the degree of overlap between the supports of two sparse signal can be estimated by measuring the sparsity degree of their sum (or difference) [10]. Finally, sparsity estimation can be used to decide whether a signal can be represented in a sparse way according to a specific basis, which can be used to select the most suitable basis allowing the sparsest representation.

### Related work

The problem of estimating the sparsity degree has begun to be recognized as a major gap between theory and practice [11–13], and the literature on the subject is very recent.

In some papers, the joint problem of signal reconstruction and sparsity degree estimation is investigated, in particular for time-varying settings. The following iterative approach is considered: given an initial upper bound for the sparsity degree, at a generic time step *t*, the signal is reconstructed and sparsity degree is estimated; such estimation is then used at time *t*+1 to assess the number of measurements sufficient for reconstruction. The seminal work [14] investigates the problem in the framework of spectrum sensing for cognitive radios and proposes an iterative method that at each time step performs two operations: (a) the signal is recovered via Lasso, and (b) the sparsity degree is estimated as the number of recovery components with magnitude larger than an empirically set threshold. The efficiency of this procedure is validated via numerical simulations.

Some authors propose sequential acquisition techniques in which the number of measurements is dynamically adapted until a satisfactory reconstruction performance is achieved [15–19]. Even if the reconstruction can take into account the previously recovered signal, these methods require to solve a minimization problem at each newly acquired measurement and may prove too complex when the underlying signal is not sparse, or if one is only interested in assessing the sparsity degree of a signal under a certain basis without reconstructing it.

In other papers, the sparsity degree estimation is only considered, which generally requires less measurements than signal reconstruction. In [13], sparsity degree is estimated through an eigenvalue-based method, for wideband cognitive radios applications. In this work, the signal reconstruction is not required, while in practice, the used number of measurements was quite large. In [20], the sparsity of the signal is lower-bounded through the numerical sparsity, i.e., the ratio between the *ℓ*_1_ and *ℓ*_2_ norms of the signal, where these quantities can be estimated from random projections obtained using Cauchy-distributed and Gaussian-distributed matrices, respectively. A limitation of this approach is that it is not suitable for adaptive acquisition since measurements taken with Cauchy-distributed matrices cannot be used later for signal reconstruction. In [21], this approach is extended to a family of entropy-based sparsity measures of kind (∥*x*∥_*q*_/∥*x*∥_1_)^*q*/(1−*q*)^ with *q*∈[0,2], for which estimators are designed and theoretically estimated in terms of limiting distributions. In [22], the authors propose to estimate the sparsity of an image before its acquisition, by calculating the image complexity. However, the proposed method is based on the image pixel values and needs a separate estimation that does not depend on the measurements. Further, in [23], the minimum number of measurements to recovery, the sparsity degree was theoretically investigated.

Finally, we notice that the problem of estimating the sparsity degree of a vector is partially connected to the problem of estimating the number of distinct elements in data streams [24, 25], which has been largely studied in the last decades due to its diverse applications. The analogy lies in the fact that the sparsity degree problem could be seen as the estimation of the number of elements distinct from zero. Moreover, many efficient algorithms to estimate the number of distinct elements are based on random hashing (see [25] for a review) to reduce the storage space, which is our concern as well. However, the problem of distinct elements considers vectors *a*=(*a*_1_,…,*a*_*n*_) with *a*_*i*_∈*Q*, where *Q* is a finite set, which is intrinsically different from our model where the signal *x* has real-valued components. Therefore, the strategies conceived for this problem cannot be applied for our purpose.

### Our contribution

In this paper, we propose a technique for directly estimating the sparsity degree of a signal from its linear measurements, without recovery. The method relies on the fact that measurements obtained by projecting the signal according to a *γ*-sparsified random matrix are distributed according to a mixture density whose parameters depend on *k*. This is an extension of the algorithm in [26], which works only in the case of noise-free, exactly *k*-sparse signals. First, we analyze the case of noise-free, exactly *k*-sparse signals as a special case, and we provide theoretical guarantees regarding the consistency of the proposed estimator and its asymptotic behavior under different regimes of the parameters *k* and *γ*. Then, we analyze the more generic case of noise, including the non-exactly sparse signals, and we propose to approximate the measurement model by a two-component Gaussian mixture model (2-GMM), whose parameters can be easily estimated via expectation-maximization (EM) techniques. In this case, we prove that there is a regime of behavior, defined by the scaling of the measurement sparsity *γ* and the sparsity degree *k*, where this approximation is accurate. An interesting property of the proposed method is that measurements acquired using a *γ*-sparsified random matrix also enable signal reconstruction, with only a slight performance degradation with respect to dense matrices [27, 28].

Some preliminary results, limited to the sparsity estimation of noisy, exactly *k*-sparse ternary signals, have appeared in [29]. In this paper, we extend the results in [29] from both a theoretical and a practical point of view, by considering any *k*-sparse signal and extending the model to non-exactly sparse signals.

### Outline of the paper

The paper is organized as follows. Section 2 presents the notation and a brief review of CS-related results. The sparsity estimation problem is formally introduced in Section 3, where we discuss the optimal estimator, whereas the main properties of the optimal estimator in the noise-free setting are outlined in Section 4. In Section 5, we introduce the proposed iterative algorithm for dealing with the noisy setting, together with some approximate performance bounds. Finally, the proposed estimators are experimentally validated in Section 6, while concluding remarks are given in Section 7.

## Preliminaries

In this section, we define some notation, we review the CS fundamentals, and we briefly discuss the use of sparsified matrices in the CS literature.

### Notation

Throughout this paper, we use the following notation. We denote column vectors with small letters, and matrices with capital letters. If $x\in \mathbb {R}^{n}$, we denote its *j*th element with *x*_*j*_ and, given *S*⊆[*n*]:={1,…,*n*}, by *x*|_*S*_, the subvector of *x* corresponding to the indices in *S*. The support set of *x* is defined by supp(*x*)={*i*∈[*n*]:*x*_*i*_≠0} and we use ∥*x*∥_0_=|supp(*x*)|. Finally, the symbol ∥*x*∥ with no subscript has always to be intended as the Euclidean norm of the vector *x*.

This paper makes frequent use of the following notation for asymptotics of real sequences $(a_{n})_{n\in \mathbb {N}}$ and $(b_{n})_{n\in \mathbb {N}}$: (i) *a*_*n*_=*O*(*b*_*n*_) for *n*→*∞* if there exists a positive constant *c*∈(0,+*∞*) and $n_{0}\in \mathbb {N}$ such that *a*_*n*_≤*cb*_*n*_ for all *n*>*n*_0_, (ii) *a*_*n*_=*Ω*(*b*_*n*_) for *n*→*∞* if there exists a constant *c*^′^∈(0,+*∞*) and $n_{1}\in \mathbb {N}$ such that *a*_*n*_≥*c*^′^*b*_*n*_ for all *n*>*n*_0_, (iii) *a*_*n*_=*Θ*(*b*_*n*_) for *n*→*∞* if *a*_*n*_=*O*(*b*_*n*_) and *a*_*n*_=*Ω*(*b*_*n*_), and (iii) *a*_*n*_=*o*(*b*_*n*_) for *n*→*∞* means that ${\lim }_{n\rightarrow \infty } |a_{n}/b_{n}| = 0$.

Given a random variable, we denote the probability density function with *f*.

### Sparse signal recovery using sparse random projections

Let $x\in \mathbb {R}^{n}$ be an unknown deterministic signal. CS [30] aims to recover a signal from a small number of non-adaptive linear measurements of the form

1 $$ y=Ax+\eta.  $$

where $y\in \mathbb {R}^{m}$ is a vector of observations, $A\in \mathbb {R}^{m\times n}$ is the sensing matrix with *m*<*n*, $\eta \in \mathbb {R}^{m}$ is an additive Gaussian noise N(0,*σ*^2^*I*_*m*×*m*_), and *I*_*m*×*m*_ is the identity matrix with *m* rows, and *m* is the columns. Since the solution to (1) is not unique, the signal is typically assumed to be sparse, i.e., it can be represented with *k* non-zero coefficients, or compressible, in the sense that it can be well approximated by a vector having only *k* non-zero coefficients. In the following, we refer to *k* as the signal sparsity degree and we denote the set of signals with exactly *k* non-zero components as $\Sigma _{k}=\{v\in \mathbb {R}^{n}:\|v\|_{0}\leq k\}$.

The literature describes a wide variety of approaches to select the sparsest solution to the affine system in (1). In particular, a large amount of work in CS investigates the performance of *ℓ*_1_ relaxation for sparse approximation.

The problem of recovery can be analyzed in deterministic settings, where the measurement matrix *A* is fixed, or in random settings in which *A* is drawn randomly from a sub-Gaussian ensemble. Past work on random designs has focused on matrices drawn from ensemble of dense matrices, i.e., each row of *A* has *n* non-zero entries with high probability. However, in various applications, sparse sensing matrices are more desirable [31]. Furthermore, sparse measurement matrices require significantly less storage space, and algorithms adapted to such matrices have lower computational complexity [32, 33]. In [27], the authors study what sparsity degree is permitted in the sensing matrices without increasing the number of observations required for support recovery.

In this paper, we consider *γ*-sparsified matrices [27], in which the entries of the matrix *A* are independently and identically distributed according to

2 $$ A_{ij}\sim\begin{cases} \mathsf{N}\left(0,\frac{1}{\gamma}\right)& \text{w.p.}\ \gamma,\\ \delta_{0}&\text{w.p.}\ 1-\gamma \end{cases}  $$

where *δ*_0_ denotes a Dirac delta centered at zero.

Since weak signal entries could be confused with noise, in [27], the support recovery is studied also as a function of the minimum (in magnitude) non-zero value of *x*:

3 $$ \lambda:=\min_{i\in\text{supp}(x)}|x_{i}|.  $$

Consequently, for a fixed *λ*>0, let us define:

4 $$ \mathcal{X}_{k}(\lambda)=\{x\in\Sigma_{k}: |x_{i}|\geq \lambda\}.  $$

For this class of signals, the following result has been proved.

#### **Theorem 1**

(Corollary 2 in [27]) Let the measurements matrix $A\in \mathbb {R}^{m}$ be drawn with i.i.d. elements from the *γ*-sparsified Gaussian ensemble. Then, a necessary condition for asymptotically reliable recovery over the signal class $\mathcal {X}_{k}(\lambda)$ is

5 $$ m\geq \max\{g_{1}(n,k,\lambda,\gamma),g_{2}(n,k,\lambda,\gamma)\}  $$

where

6 $$ {\begin{aligned} g_{1}(n,k,\lambda,\gamma)\geq \frac{\log{n\choose k}-1}{\frac{1}{2}\gamma k\log\left(1+\lambda^{2}k/\sigma^{2}\right)+\frac{1}{2}\log\left(2\pi \mathrm{e}\left(\gamma k+\frac{1}{12}\right)\right)} \end{aligned}}  $$

7 $$ g_{2}(n,k,\lambda,\gamma)\geq \frac{\log{(n-k+1)-1}}{\frac{1}{2}\gamma\log\left(1+\lambda^{2}/\left(\sigma^{2}\gamma\right)\right).}  $$

In particular, Theorem 1 says that if *γk*→*∞* as *n*→*∞*, then the number of measurements is of the same order as that for dense sensing matrices. In sharp contrast, if *γk*→0 sufficiently fast as *n*→*∞*, then the number of measurements of any decoder increases dramatically. Finally, if *γk*=*Θ*(1) and *λ*^2^*k*=*Θ*(1), then at least max{*Θ*(*k* log(*n*/*k*)),*Θ*(*k* log(*n*−*k*)/ log*k*)} measurements are necessary for estimating the support of the signal.

Several recovery algorithms are based on the use of sparse sensing matrices. In particular, count-minute sketch algorithms need about 10 to 15 times more measurements than *ℓ*_1_-decoding and sparse matching pursuit needs about half of the measurements of count-min sketch. Other sketch algorithms include [34] that can be as accurate as *ℓ*_1_ decoding with dense matrices under the condition *γk*=*Θ*(1) with the same order of measurements.

## Sparsity estimation problem: mathematical formulation

Our goal is to estimate *k* from the measurements *y*=*Ax*, where *A* is a *γ*-sparsified matrix, without the burden of reconstructing *x*. Specifically, we aim at providing conditions on the triplet (*n*,*m*,*k*) as well as on *x* and *A* under which the estimation of signal sparsity is accurate with high probability. The theoretical results that we provide also hold true for high dimensional settings, that is, (*n*,*m*,*k*), and are allowed to tend to infinity.

Given a rule for computing estimates of the signal sparsity, we will measure the error between the estimate $\widehat {k}(m,n)$ and the true sparsity degree *k* using the relative error:

8 $$ e\left(k,\widehat{k}\right):={\left|k-\widehat{k}\right|}/{k}.  $$

We say that the sparsity estimator $\widehat {k}$ is asymptotically *weakly consistent* when $e\left (k,\widehat {k}\right)$ converges in probability to 0 as *m*,*n*→*∞*. If we replace convergence in probability with almost sure convergence, then the estimator is said to be *strongly consistent*.

If the signals are not exactly sparse but compressible, i.e., they admit a representation with few large components in magnitude; in CS literature, the recovery guarantees are expressed in terms of the sparsity degree of the best-*k* approximation [30, 35] defined as follows

9 $$\widehat{x}_{k}=\underset{{z\in\Sigma_{k}}}{\mathrm{argmin\,}}\|x-z\|. $$

For this reason, the sparsity of a not exactly sparse signal is defined as the number of components containing most of the energy up to a relative error *τ*

10 $$ {k}_{\tau}=\min\{s\in[n]: \|x-\widehat{x}_{s}\|^{2}\leq \tau \|x\|^{2}\}.  $$

Then, defining $e=x-\widehat {x}_{k_{\tau }}$, we write

11 $$ y=Ax=A\left(\widehat{x}_{k_{\tau}}+e\right)=A\widehat{x}_{k_{\tau}}+\eta  $$

where *η*=*Ae*. It should be noticed that each component *η*_*i*_ is distributed as is a mixture of Gaussians. We make the following approximation: *η*_*i*_∼N(0,*σ*^2^) with

12 $$\begin{array}{*{20}l} \sigma^{2}&=\mathbb{E}\left[\eta^{2}_{i}\right]=\|e\|^{2}\leq\tau\|x\|^{2}. \end{array} $$

In the noiseless case, by linearity of expectation, we have

$${\begin{aligned} \mathbb{E}\left[\frac{1}{m}\sum_{i=1}^{m} y_{i}^{2}\right] &= \frac{1}{m}\sum_{i=1}^{m}\mathbb{E}\left[y_{i}^{2}\right]\\ &= \frac{1}{m}\sum\limits_{i=1}^{m}\mathbb{E}\left[\sum\limits_{j=1}^{n}A_{ij}^{2}x_{j}^{2}+2\sum_{j< k}A_{ij}A_{ik}x_{j}x_{k}\right] \end{aligned}} $$

from which

$${\begin{aligned} \frac{1}{m}\sum_{i=1}^{m} \mathbb{E}\left[y_{i}^{2}\right] &= \frac{1}{m}\sum_{i=1}^{m}\sum_{j=1}^{n}\mathbb{E}\left[A_{ij}^{2}\right]x_{j}^{2}\\ & \quad +2\sum_{j< k}\mathbb{E}\left[A_{ij}\right]\mathbb{E}\left[A_{ik}\right]x_{j}x_{k}=\|x\|^{2} \end{aligned}} $$

where the last inequality follows from $\mathbb {E}\left [A_{ij}\right ]=0$ and $\mathbb {E}\left [A_{ij}^{2}\right ]=1$ for all *i*,*j*. Then, the model describing the measurements can be approximated by (1) with *σ*^2^≈*τ*∥*y*∥^2^/*m*. We underline that this argument is true for all sensing matrices drawn from the ensemble in (2), and at this time, we do not make any additional assumption on the number of measurements.

Given (*y*,*A*) and assuming that the perturbation is additive Gaussian, the ML estimator of the signal sparsity can be obtained via the following exhaustive search:

13 $$ \underset{{s\in[n]}}{\text{argmin}}\left\{\min_{T\subseteq [n]:|T|=s,\{x:\text{supp}(x)=T\}}\|Ax-y\|^{2}_{2}\right\}.  $$

However, this optimization problem is NP hard and the search of the solution requires an exponential time in the signal length *n* (one optimization problem for all subsets of [ *n*] of size *s* and for all *s*, which amounts to $\sum _{s=1}^{n} {n\choose s} = 2^{n} -1$).

Given supp(*x*), if *A* is chosen from the ensemble of *γ*-sparsified matrices, any measurement

14 $$ y_{i}=\sum_{j\in[n]}A_{ij}x_{j}+\eta_{i}  $$

is a random variable whose density function is a mixture of Gaussians with 2^*k*^ components. The result follows from the following argument. Let *S* be the overlap between the support of *i*th row of *A* and supp(*x*). It is easy to see that given *S* then *y*_*i*_∼N(0,*α*_*S*_) where *α*_*S*_=∥*x*_*S*_∥^2^/*γ*+*σ*^2^. Without any further assumption, taking into account all possible overlaps between the support of the *i*th row of *A* and support of the signal *x* with cardinality *s*≤*k*, we can have in principle $\sum _{s\leq k}{k\choose s}=2^{k}$ different type of Gaussians. We conclude that *y*_*i*_ is a Gaussian mixture with 2^*k*^ components. If the non-zero elements of the signal have all equal values in magnitude, then the number of components of the Gaussian mixture reduces dramatically to *k*. Given the set of *m* independent and identically distributed samples *y*=(*y*_1_,…,*y*_*m*_)^⊤^, the sparsity estimation can be recast into the problem of evaluating the number of mixture components and parameters. However, also in the simple case where *k* is known, the estimation of the finite mixture density function does not admit a closed-form solution, and the computational complexity is practically unfeasible.

## Method: noise-free setting

In this section, we show that in the absence of noise (i.e., ∥*η*∥=0), ∥*y*∥_0_ is a sufficient statistic for the underlying parameter *k*. We show that the performance of the proposed estimators of the sparsity degree depends on the *SNR*=*λ*^2^*k*/*σ*^2^ and that the traditional measure ∥*x*∥^2^/*σ*^2^ has no significant effect in the estimation of the sparsity degree.

Even in the absence of noise, since *A* is chosen from the ensemble of *γ*-sparsified matrices, any measurement $y_{i}=\sum _{j\in [n]}A_{ij}x_{j} $ is still a random variable. The ML solution provides the following estimator of the signal sparsity.

### **Proposition 1**

Let us define 
15

Then, the ML estimate of the signal sparsity is

16 $$ \widehat{k}_{o}=\frac{\log\left(1-\frac{\|\omega^{\star}\|_{0}}{m}\right)}{\log(1-\gamma)}.   $$

The estimator derived in proposition 1 has already been proposed in [26] for estimating the degree of sparsity. In the following, we will denote the estimator in (16) as *oracle estimator* since it is equivalent to estimating *k* in the presence of an oracle who knows which entries in *y* are only due to noise. In our analysis, we prove that the oracle estimator is asymptotically strongly consistent, i.e, with the property that as the number of measurements increases indefinitely, the resulting sequence of estimates converges almost surely to the true sparsity degree (see Theorem 2). This means that the density functions of the estimators become more and more concentrated near the true value of the sparsity degree.

Given a sequence of events $\{E_{m}\}_{m\in \mathbb {N}}$, we denote with $\limsup _{m\rightarrow \infty }E_{m}$ the set of outcomes that occur infinitely many times. More formally,

17 $$ \limsup_{m\rightarrow\infty}E_{m}=\bigcap_{m=1}^{\infty}\bigcup_{\ell\geq m}^{\infty}E_{\ell}  $$

### **Theorem 2**

Let *p*_*k*_=1−(1−*γ*)^*k*^, then

18 $$ \mathbb{P}\left(e\left(\widehat{k}_{o},k\right)>\epsilon\right)\leq 2\mathrm{e}^{-2m\xi_{k}^{2}},  $$

where $\xi _{k}={(1-p_{k})}\left (1-\mathrm {e}^{\epsilon \log (1-p_{k})}\right).$ Moreover, let *E*_*m*_ be the sequence of events

19 $$ E_{m}=\left\{e(\widehat{k}_{o},k)\geq\frac{\log\left(1-\frac{\sqrt{\rho}}{1-p_{k}}\sqrt{\frac{\log m}{m}}\right)}{\log (1-p_{k})}\right\}  $$

with $m\in \mathbb {N}$ and *ρ*>1/2, then

20 $$ \mathbb{P}\left(\limsup_{m\rightarrow\infty}E_{m}\right)=0.  $$

### **Remark 1**

From Theorem 2, we deduce that almost surely (i.e., with probability *1*) the relative error between the estimated sparsity and the true value of the sparsity degree is

21 $$e \left(\widehat{k}_{o},k\right)= O\left(\sqrt{\frac{{\log m}}{m}}\right) $$

for all but finitely many *m*.

### Asymptotic analysis of ML estimator

We analyze now the performance of the oracle estimator in the large system limit, when *n*,*k*,and *m* tend to infinity. Since we are dealing with sparse signals, we assume that the sparsity degree *k* scales at most linearly in the signal length, i.e., the relative sparsity *k*/*n*≤*ρ* is kept bounded with *ρ*≪1. The following theorem shows sufficient conditions on the number of measurements for weak consistency in different regimes of behavior, defined by the scaling of the measurement sparsity *γ* and the signal sparsity *k*.

#### **Theorem 3**

Let *ψ*(*k*)=*γk*=*o*(*k*) as *k*→*∞* and define the function *g*(*k*) as follows:

- if *ψ*(*k*)→*∞* as *k*→*∞*, then *g*(*k*)=*Ω*(e^2*ψ*(*k*)^);
- if *ψ*(*k*)=*Θ*(1) as *k*→*∞*, then *g*(*k*)→*∞* for *k*→*∞*;
- if *ψ*(*k*)=*o*(1) as *k*→*∞*, then *g*(*k*)=*Ω*(*ψ*(*k*)^−2(1+*ε*)^), for any *ε*>0.

If the number of measurements is such that $\frac {m}{\log m}\geq g(k)$, then

22 $$\mathbb{P}\left(e\left(\widehat{k}_{o},k\right)\geq \epsilon_{k}\right)\stackrel{k\rightarrow\infty}{\longrightarrow}0,  $$

where

23 $$\epsilon_{k}=\frac{\log\left(1-\frac{\sqrt{\rho}}{1-p_{k}}\sqrt{\frac{\log m}{m}}\right)}{\log (1-p_{k})}\stackrel{k\rightarrow\infty}{\longrightarrow}0 $$

for some constant *ρ*>1/2.

In the following theorem, we show that, under stricter conditions, strong consistency is also ensured.

#### **Theorem 4**

Let *ψ*(*k*)=*γk* and define the function *g*(*k*) as in Theorem 3. If the number of measurements is such that

24 $$m\geq\max\left\{k,\min\left\{\ell\in\mathbb{N}:\frac{\ell}{\log \ell}\geq g(k)\right\}\right\} $$

then

25 $$\mathbb{P}\left(\limsup_{k\rightarrow\infty}e\left(\widehat{k}_{o},k\right)= 0\right){=}1.  $$

#### **Remark 2**

Theorems 3 and 4 characterize the regimes in which measurement sparsity begins to improve the estimation of the signal sparsity. The function *ψ*(*k*)=*γk* represents the average number of non-zeros in each row of *A* that align with the support of the signal *x*. This analysis reveals three cases of interest, corresponding to whether measurement sparsity has no effect, a small effect, or a significant effect on the number of measurements sufficient for asymptotic consistency. If *ψ*(*k*)=*Θ*(1) as *k*→*∞*, then *m*=*Ω*(*k*) measurements are sufficient for the concentration result. In sharp contrast, if *ψ*(*k*)→*∞* as *k*→*∞*, then the number of measurements guaranteeing the asymptotic consistency is exponential in *ψ*(*k*), meaning that, in order to be sure to get an unbiased estimator with *k* measurements, we need $\psi (k)\leq \frac {1}{2}\left (\log k-\log (\log k)\right)$. If *ψ*(*k*)→0, then the condition $\psi (k)\geq \sqrt [2+\epsilon ]{\frac {\log (k)}{k}}$ with *ε*>0 is sufficient to get an unbiased estimator with *k* measurements.

#### **Remark 3**

Theorems 3 and 4 suggest that in order to obtain a good estimation of the sparsity degree, we need sufficiently sparse matrices, but not too sparse. On the other hand, at the same time, the use of sparser matrices requires more measurements for a successful recovery of the signal. If we combine the results obtained in Theorems 3 and 4 with those provided in Theorem 1, we notice there is a large range for *γ* (provided by $c\leq \psi (k)\leq \frac {1}{2}\left (\log k-\log (\log k)\right)$ with *c*>0) where both sparsity estimation and the recovery can be successful. We will provide more details on how to choose *γ* for specific applications in Section 6.

The proofs of Theorems 3 and 4 are postponed to the Appendix.

## Method: noisy setting

As already noticed, in the generic noisy setting, the estimation of signal sparsity via an exhaustive ML is unfeasible. A possible approach is to resort to the well-known EM algorithm [36]. This algorithm can find ML solutions to problems involving observed and hidden variables, and in the general, setting is known to converge to a local maximum of the likelihood.

In this section, we prove that, under suitable conditions, the distribution of the measurements can be well approximated by a two-component Gaussian mixture that can be easily learned by EM algorithm. Finally, we show that the case of not-exactly sparse signals can be well approximated by the same model.

### 2-GMM approximation for large system limit

Our main goal is to show that the Gaussian mixture model that describes the measurements can be simplified in the large system limit as *n*,*k*→*∞*. The next theorem reveals that there is a regime of behavior, defined by the scaling of the measurement sparsity *γ* and the signal sparsity *k*, where the measurements can be approximately described by a two-component Gaussian mixture model (2-GMM). We state this fact formally below. Recall that, given two density functions *f*,*g*, their Kolmogorov distance is defined as

26 $$ \|f-g\|_{\mathrm{K}}=\sup_{t\in\mathbb{R}}\left|\int_{-\infty}^{t}f(\zeta)\mathrm{d}\zeta-\int_{-\infty}^{t}g(\zeta)\mathrm{d}\zeta\right|.  $$

#### **Theorem 5**

Let supp(*x*)=*k* and *ϕ*(*ζ*|*σ*^2^) be the probability density function of a normally distributed random variable with expected value 0 and variance *σ*^2^, i.e.,

$$\phi\left(\zeta|\sigma^{2}\right)=\frac{1}{\sigma\sqrt{2\pi}}\mathrm{e}^{-\frac{\zeta^{2}}{2\sigma^{2}}}. $$

Given a set *S*, let *α*_*S*_=∥*x*_*S*_∥^2^/*γ*+*σ*^2^ and *p*_*S*_=(1−*γ*)^*k*−|*S*|^*γ*^|*S*|^. Let us consider the density functions (the subscript is to emphasize the dependence on parameter *k*)

27 $$ f_{k}(\zeta)=\sum_{S\subseteq\text{supp}(x)}p_{S}\phi(\zeta|\alpha_{S})  $$

28 $$ f^{\textrm{2-GMM}}_{k}(\zeta)=(1-p_{k})\phi\left(\zeta|\sigma^{2}\right)+p_{k}\phi\left(\zeta\Big|\sigma^{2}+\frac{\|x\|^{2}}{p_{k}}\right).  $$

Then, there exists a constant $C\in \mathbb {R}$ such that

29 $$ \left\|f_{k}-f^{\textrm{2-GMM}}_{k}\right\|_{\mathrm{K}}\leq\frac{C\gamma^{2}}{\lambda^{4}}\left(\frac{\sum_{i=1}^{n}x_{i}^{4}}{\gamma}+\sum_{i\neq j}x_{i}^{2}x_{j}^{2}-\frac{\|x\|^{4}}{p_{k}}\right).  $$

The proof of Theorem 5 is postponed to the Appendix. As a simple consequence, we obtain that, under suitable conditions, the approximation error depends on *ψ*(*k*).

#### **Corollary 1**

Let *ψ*(*k*)=*γk* and $f_{k}, f_{k}^{\textrm {2-GMM}}$ be the sequence of density functions defined in (27) and (28). Then, there exists a constant $C^{\prime }\in \mathbb {R}$ such that

30 $$\begin{array}{*{20}l} \left\|f_{k}-f^{\textrm{2-GMM}}_{k}\right\|_{\mathrm{K}}\leq C^{\prime}\left(\frac{\lambda_{\max}}{\lambda_{\min}}\right)^{4}\left(\psi(k)+\psi(k)^{2}\right). \end{array} $$

with *C*^′^≈0.03, $\lambda _{\max }=\max \limits _{i:x_{i}\neq 0}|x_{i}|$, $\lambda _{\min }=\min \limits _{i:x_{i}\neq 0}|x_{i}|$.

Corollary 1 shows that the error in the approximation can be controlled by parameter *ψ*(*k*). Some considerations are in order. Consider for example a *k*-sparse signal with all non-zero components equal in modulus, i.e., with *λ*_max_=*λ*_min_. Then, the bound reduces to $ \left \|f_{k}-f^{\mathrm {2-GMM}}_{k}\right \|_{\mathrm {K}}\leq C\left (\psi (k)+\psi (k)^{2}\right)$. We can see that if *ψ*(*k*)=*γk*→0, then the Kolmogorov distance goes to zero. However, as suggested by Theorem 1, we expect to need more measurements *m* to perform a good estimation of the sparsity degree. The best regime is when *ψ*(*k*)=*Θ*(1) as *k*→*∞*: in that case, the distance remains bounded and we expect that a number of measurements proportional to *k* is sufficient for the sparsity estimation (suppose, for example, that *γ*=3/*k*. Then, we expect $\left \|f_{k}-f^{\mathrm {2-GMM}}_{k}\right \|_{\mathrm {K}}< 0.36$). For signals with *λ*_max_≠*λ*_min_ similar considerations can be done if *λ*_max_ and *λ*_min_ scale similarly as a function of *k*.

### Sparsity estimation via EM

Using the approximation in Theorem 5, we recast the problem of inferring the signal sparsity as the problem of estimating the parameters of a two-component Gaussian mixture, whose joint density function of $y\in \mathbb {R}^{m}$ and hidden class variables *z*∈{0,1}^*m*^ is given by

31 $$ f(y_{i},z_{i}|\alpha,\beta,p)=(1-p)(1-z_{i})\phi(y_{i}|\alpha)+{pz}_{i}\phi(y_{i}|\beta)  $$

with *i*∈ [*m*]. Starting from an initial guess of mixture parameters *α*(0),*β*(0),and*p*(0), the algorithm that we propose (named EM-Sp and summarized in Algorithm 1) computes, at each iteration $t\in \mathbb {N}$, the posterior distribution

32 $$ \pi_{i}(t)=\mathbb{P}(z_{i}=1|\alpha{(t)},\beta{(t)},p(t))  $$

(E-Step) and re-estimates the mixture parameters (M-Step) until a stopping criterion is satisfied. Finally, the estimation of the signal sparsity is provided by

33 $$ \widehat{k}=\log(1-p_{\text{final}})/\log(1-\gamma).  $$

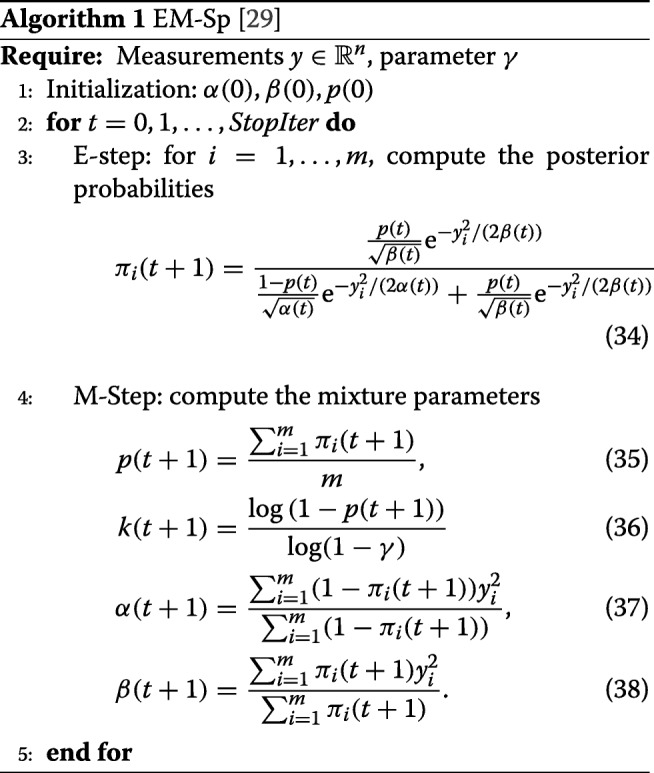


The sequence of signal sparsity estimations *k*(*t*) generated by Algorithm 1 converges to a limit point. For brevity, we omit the proof, which can be readily derived from standard convergence arguments for dynamical systems [37]. In the following, we will denote Algorithm 1 as EM-Sparse (EM-Sp).

### The Cramér-Rao bound for 2-GMM

The Cramér-Rao (CR) bound is a popular lower bound on the variance of estimators of deterministic parameters. Given a parameter *ξ* and an unbiased estimator $\widehat {\xi }$, let *f*(*x*;*ξ*) be the likelihood function. The CR bound is given by

39 $$\text{CR}(\widehat{\xi})=\frac{1}{\mathbb{E}\left(\frac{\partial \log(f(x; \xi))}{\partial \xi}\right)^{2}}  $$

that is, the inverse of the Fisher information.

The EM-Sp algorithm, for measurements that can be exactly modeled as a 2-GMM, and for a large number of measurements, would be asymptotically optimal and unbiased and achieve a performance very close to the CR bound. However, because of the presence of noise in the data and the approximation of the 2-GMM model, we expect that the estimator provided by EM-Sp algorithm will be biased. A theoretical analysis of the bias in terms of these two factors is hard to carry out. In the following, we analyze the performance of EM-Sp in the estimation of the 2-GMM parameters via the CR bound, which gives us an indication of how much the non-idealities of the model affect the performance of the proposed estimator.

Let us consider a 2-GMM framework, in which two zero-mean Gaussians with known variances *α* and *β* are given, and let us call *p* the mixture parameter. The likelihood function is *f*(*x*;*p*)=(1−*p*)*ϕ*(*x*|*α*)+*pϕ*(*x*|*β*), and

40 $$ \text{CR}(\widehat{p})=\frac{1}{\mathbb{E}\left(\frac{\phi(x|\beta)-\phi(x|\alpha)}{(1-p)\phi(x|\alpha)+p\phi(x|\beta)}\right)^{2}}  $$

is the CR bound for the ML estimator $\widehat {p}$ of *p*. The stochastic mean cannot be computed in a closed form, but can be approximated with a Monte Carlo method.

CR$(\widehat {p})$ represents a benchmark to evaluate the accuracy of our estimation of *p* via EM-Sp, as will be done in Section 6.2.

## Results and discussion

In this section, we illustrate the performance of the proposed estimators through extensive numerical simulations^1^. We present experiments both in the noise-free setting and in non-ideal settings, where signals are not exactly sparse or measurements are affected by noise. Finally, an application where sparsity estimation improves the efficiency of signal recovery in CS is proposed.

### Noise-free measurements

We start testing signals that are exactly sparse and measurements that are not affected by additive noise.

**Fig. 1:**
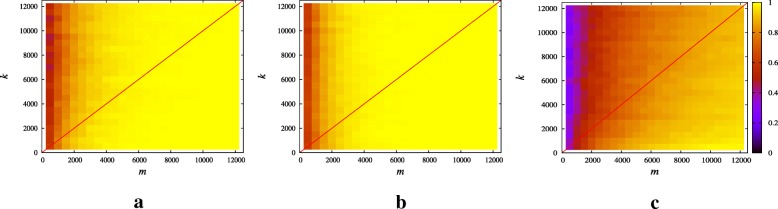
Noise-free setting: empirical probability of success sparsity estimation as a function of sparsity degree *k* and number of measurements. **a**$\psi (k)=\frac {1}{2}\text {log}\left (\frac {k}{\text {log}\ k}\right)$**b** *ψ*(*k*)=1**c**$\psi (k)=\sqrt [3]{\frac {\text {log}\ k}{k}}$

We evaluate the estimation accuracy in terms of empirical probability of correct estimation: a run is considered successful if $e\left (k,\widehat {k}\right)<5\times 10^{-2}$ where $\widehat {k}$ is the estimated sparsity. In Fig. 1, we show results averaged over 1000 random instances, obtained by generating different sensing matrices from the *γ*-sparsified Gaussian ensemble. We underline that the values of the non-zero entries of the signals (which are drawn from a standard Gaussian distribution for this experiment) do not affect the performance in the noise-free case. Similarly, the length *n* of the signal plays no role in the estimation (see Proposition 1).

The empirical probability of correct estimation is studied as a function of *m* and *k* for three different regimes of parameter *ψ*(*k*) defined in Remark 2 (see Fig. 1) :

- $\psi (k)=\frac {1}{2}(\log (k)-\log (\log k))$;
- *ψ*(*k*)=1;
- $\psi (k)=\sqrt [3]{{\log k}/{k}}$.

According to Theorem 3 (see also Remark 2), when *m*≥*k*, the relative error between the estimated sparsity and the true value of the sparsity degree tends to zero almost surely (i.e., with probability 1). This can be appreciated also in the numerical results in Fig. 1, where the line *m*=*k* is drawn for simplicity. Moreover, we can see that for any fixed *k*, the error decreases when *m* increases.

### Noisy measurements

In the second experiment, we show the performance of the EM-Sp algorithm when measurements are noisy according to the model proposed in (1) and we compare to the numerical sparsity estimator [20]. In order to have a fair comparison, we perform this test on ternary signals in {−*λ*,0,*λ*}^*n*^ for which sparsity and numerical sparsity coincide. We then consider random sparse signals with non-zero entries uniformly chosen in {*λ*,−*λ*}, $\lambda \in \mathbb {R}$, and *SNR*=*λ*^2^*k*/*σ*^2^ (see definition in Section 3). Moreover, we set *ψ*(*k*) constant in order to focus on the effects of the additive noise in the estimation.

We remark that we compare only to [20] because, as illustrated in Section 1.1, the other proposed algorithms for sparsity degree estimation are based on signal reconstruction [14] (requiring a larger number of measurements and increased complexity, which would give an unfair comparison) or are conceived for very specific applications [13, 22].

**Fig. 2:**
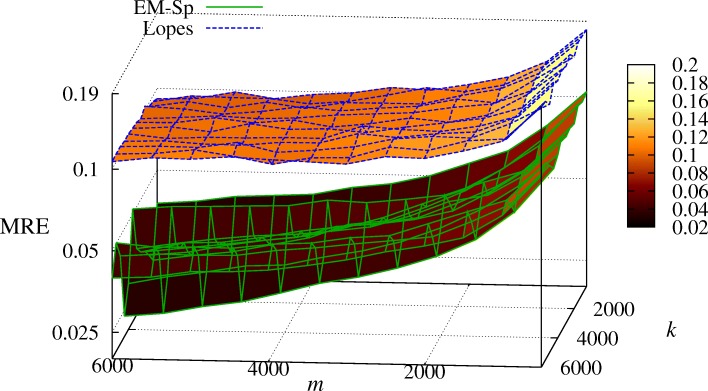
Experiment 2: MRE (in log-scale) of EM-Sp and Lopes’s estimator as a function of the sparsity degree *k* and the number of measurements *m*, *SNR*=10 dB

In Fig. 2, we show the mean relative error (MRE) defined as

41 $$ \text{MRE}=\mathbb{E}\left[e\left(k,\widehat{k}\right)\right]  $$

for different values of *k* and *m* in settings with *SNR*=10 dB and *ψ*(*k*)=1/10. We appreciate that, in the considered framework, EM-Sp always outperforms the method based on the numerical sparsity estimation.

**Fig. 3:**
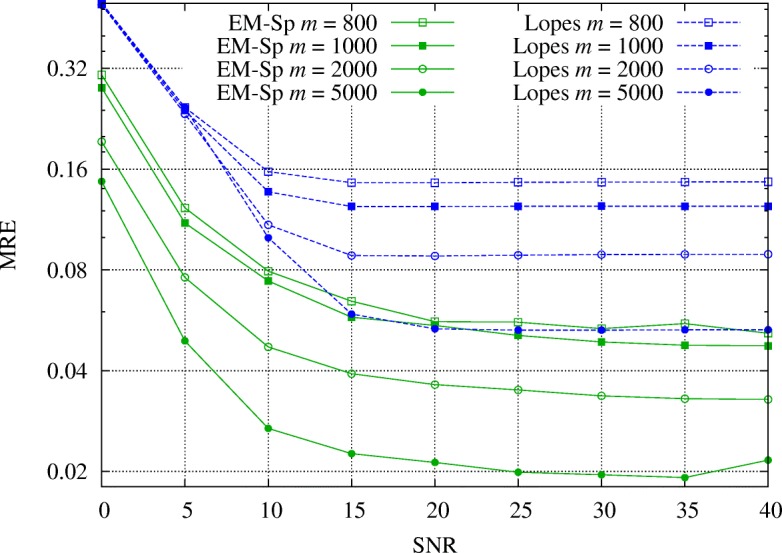
Experiment 2: MRE (in log-scale) of EM-Sp and numerical sparsity estimator as a function of the *SNR*, for different *m*’s, *k*=1000

In Fig. 3, we set *k*=1000, *ψ*(*k*)=1/3, and we vary the *SNR* from 0 to 40 dB, while *m*∈{800,1000,2000,5000}. Again, we see that EM-Sp outperforms [20]. We specify that a few tens of iterations are sufficient for the convergence of EM-Sp.

**Fig. 4:**
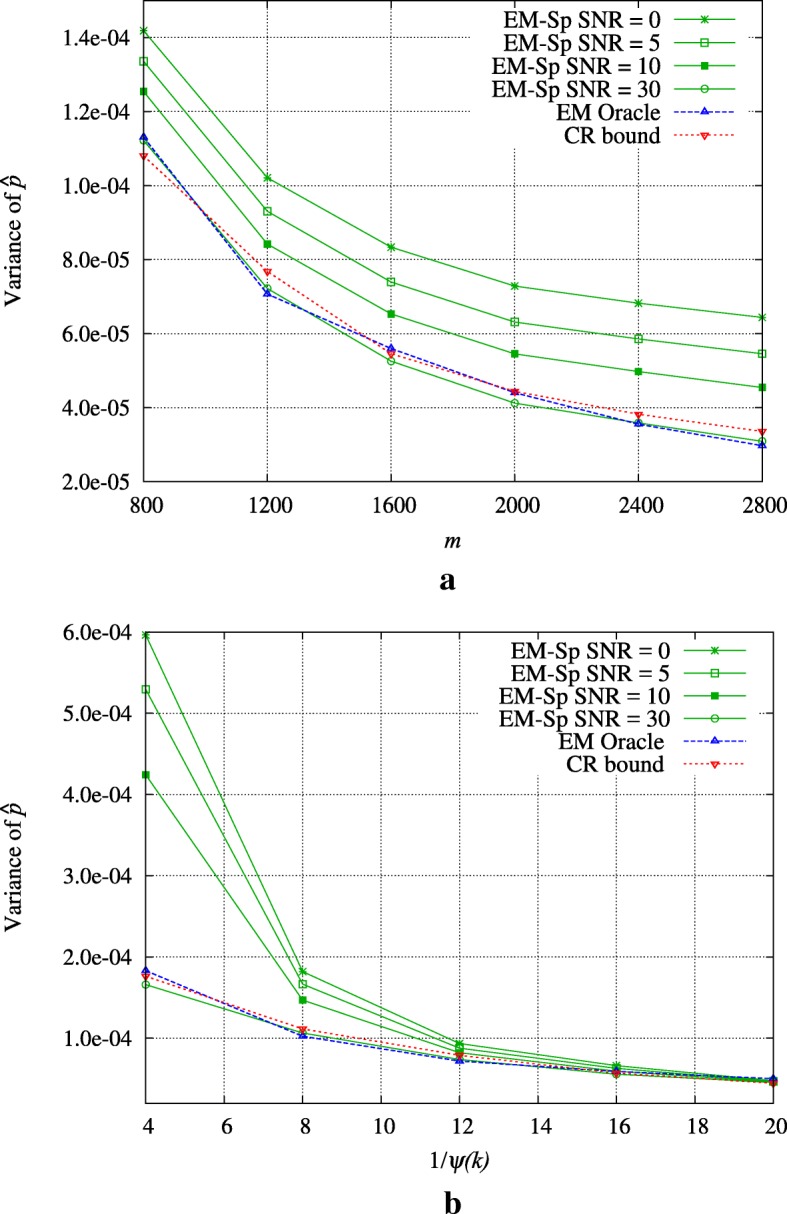
Noisy setting: comparison with oracle EM and Cramér-Rao (CR) bound. **a** *k*=1000,*ψ*(*k*)=1/10**b** *k*=*m*=1000

Finally, we compare the performance of EM-Sp with an oracle estimator designed as follows: we assume to know exactly the variances *α* and *β* and we generate measurements *y*_*i*_ distributed according to 2-GMM (1−*p*)*ϕ*(*y*_*i*_|*α*)+*pϕ*(*y*_*i*_|*β*), for *i*=1,…,*m*; given the sequence *y*, *α*, and *β*, we then compute the ML estimate of *p* via EM. We name this estimator EM oracle. Comparing the estimates of *p* of EM-Sp and EM oracle, we can check if our 2-GMM approximation is reliable. We clearly expect that EM oracle performs better, as the measurements are really generated according to a 2-GMM, and also the true *α* and *β* are exploited. However, our results show that the 2-GMM approximation is trustworthy. In Fig. 4, we depict the sample variance of the estimator $\widehat {p}$ of *p* (obtained from 1000 runs) of EM-Sp and EM oracle. We show also the CR bound (see Section 5.3), which represents a performance lower bound for the estimation of *p*. As explained in Section 5.3, the stochastic mean required in the CR bound for 2-GMM cannot be analytically computer and is here evaluated via Monte Carlo.

In both graphs of Fig. 4, we set *k*=1000, *m*=*k* in Fig. 4a, and *ψ*(*k*)=1/10 in Fig. 4b. We notice that in the considered regimes, EM oracle and CR bound are very close and not really affected by the *SNR*. Regarding EM-Sp, we observe that (a) keeping *k*,*γ* fixed, EM-Sp gets closer to the optimum as the *SNR* increases; and (b) keeping *k*,*m* fixed, we can find an optimal *γ* that allows us to get very close to the optimum.

### Compressibility of real signals

In this section, we test our EM-Sp algorithm to evaluate the compressibility of real signals. Specifically, we consider images which are approximately sparse in the discrete cosine transform (DCT) domain, that is, they are well represented by few DCT coefficients. Our aim is to estimate the number *k* of significant DCT coefficients. More precisely, we seek the minimum *k* such that the best-*k* approximation $\widehat {x}_{k}$ has a relative error smaller approximately *τ*, namely ${\left \|\widehat {x}_{k}-x \right \|_{2}^{2}}\leq \tau {\left \|x \right \|_{2}^{2}}$. Since DCT coefficients of natural images usually have a power-law decay of the form *x*_*i*_≈*c*/*i*, the following approximation holds

42 $$ \frac{\left\|\widehat{x}_{k}-x \right\|_{2}^{2}}{\left\|x \right\|_{2}^{2}}\approx \frac{\int_{k}^{n} x^{-2}\mathrm{d}x}{\int_{1}^{n} x^{-2}\mathrm{d}x}\propto k^{-1}  $$

and since according to theoretical derivation *γ*∝1/*k*, we fix *γ*∝*τ*. Tuning *γ* proportionally to *τ* allows to adapt better the sparsity of the sensing matrix to the expected signal sparsity: for larger *τ*’s, we expect smaller *k*^′^*s*, which call for larger *γ* to have the sufficient matrix density to pick the non-zero entries.

In the proposed experiments, we fix *γ*=*cτ* with *c*=5·10^−2^ and we initialize $\pi _{i}(0)=\frac {1}{2}$ for all *i*=1,…,*m*, while we set *β* and *α* respectively as the signal energy and the noise energy (namely, the error of the best-*k* approximation), evaluated from the measurements: $\beta =\frac {\left \|y\right \|_{2}^{2}}{m}$ and *α*=*τβ*.

**Fig. 5:**
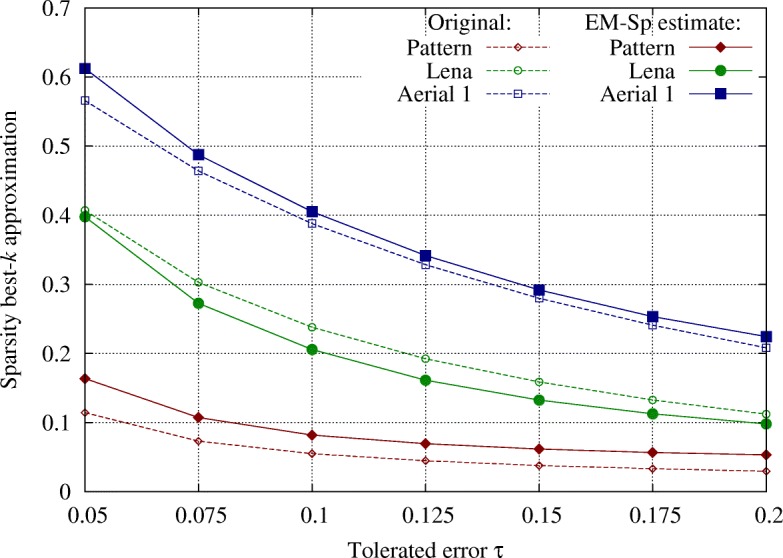
Experiment 3: compressibility of real signals, with non-exactly sparse representations. We estimate *k* such that $\frac {\left \|\widehat {x}_{k}-x \right \|_{2}^{2}}{\left \|x \right \|_{2}^{2}}<\tau $, where *x* and $\widehat {x}_{k}$ respectively are the non-exactly sparse representation and its best-*k* approximation

**Fig. 6:**
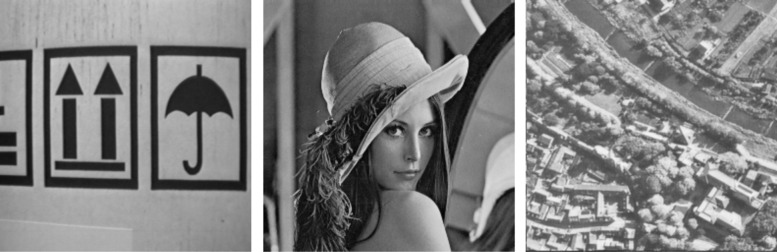
Non-exactly sparse signals: images with approximately sparse DCT representation: pattern, Lena, and aerial

In Fig. 5, we depict the results on three *n*=256×256 images (shown in Fig. 6) represented in the DCT basis (DCT is performed on 8×8 blocks). Specifically, we show original and estimated sparsity (the *y*-axis represents the ratio *k*/*n*), averaged on 100 random sensing matrices. The images have been chosen with different compressibilities, to test our method in different settings. We appreciate that for all the images and across different values of *τ*, we are able to estimate *k* with a small error. This experiment shows then that EM-Sp can be practically used to estimate the compressibility of real signals.

### Sparsity estimation for signal recovery

We have already remarked that the knowledge of the sparsity degree *k* is widely used for signal recovery in CS. In this section, we consider CoSaMP [9], an algorithm which can recover a sparse signal (exactly or with a bounded error, in the absence and in the presence of noise, respectively) if a sufficient number of measurements *m* is provided, and assuming the knowledge an upper bound *k*_max_ for *k*. Our aim is to show that EM-Sp can be used as a pre-processing for CoSaMP when *k* is not known; specifically, we estimate *k* to design the number of measurements necessary for CoSaMP recovery. Subsequently, we denote this joint procedure as EM-SP/CoSaMP.

We compare CoSaMP with EM-Sp/CoSaMP in the following setting. We consider a family $\mathcal {S}$ of signals of length *n*=1600 and (unknown) sparsity *k*∈{20,200} (then, *k*_max_=200). The value of *k* and the position of the non-zero coefficients are generated uniformly at random, and the non-zero values are drawn from a standard Gaussian distribution. Since *k* is not known, the number of measurements needed by CoSaMP has to be dimensioned on *k*_max_: assuming *SNR*=30 dB, from the literature, we get that *m*_*C*_=4*k*_max_ are sufficient to get a satisfactory recovery using dense Gaussian sensing matrices. In our specific setting, we always observe a mean relative error MRE$_{\text {rec}} =\left \|x-\widehat {x}\right \|_{2}/\left \|x\right \|_{2}<5.5\times 10^{-2}$ (for each *k*∈{20,200}, 100 random runs have been performed).

We propose now the following procedure.

1. First sensing stage and sparsity estimation: we take *m*_*S*_≪*m*_*C*_ measurements via *γ*-sparsified matrix in (2), and we provide an estimate $\widehat {k}$ of *k* using Algorithm ??.
2. Second sensing stage and recovery: we add a sufficient number of measurements (dimensioned over $\widehat {k}$) and then perform CoSaMP recovery.

Specifically, the following assessments have been proved to be suitable for our example:

- We estimate *k* with EM-Sp from $m_{S}=\frac {k_{\max }}{2}$ sparsified measurements, with *γ*=6/*k*_max_;
- Since underestimates of *k* are critical for CoSaMP, we consider $\widehat {k}$ equal to 2 times the estimate provided by EM-Sp;
- We add $m_{A}=4\widehat {k}$ Gaussian measurements, and we run CoSaMP with the so-obtained sensing matrix with *m*_*S*_+*m*_*A*_ rows. When *m*_*S*_+*m*_*A*_>*m*_*C*_, we reduce the total number of measurements to *m*_*C*_.

**Fig. 7:**
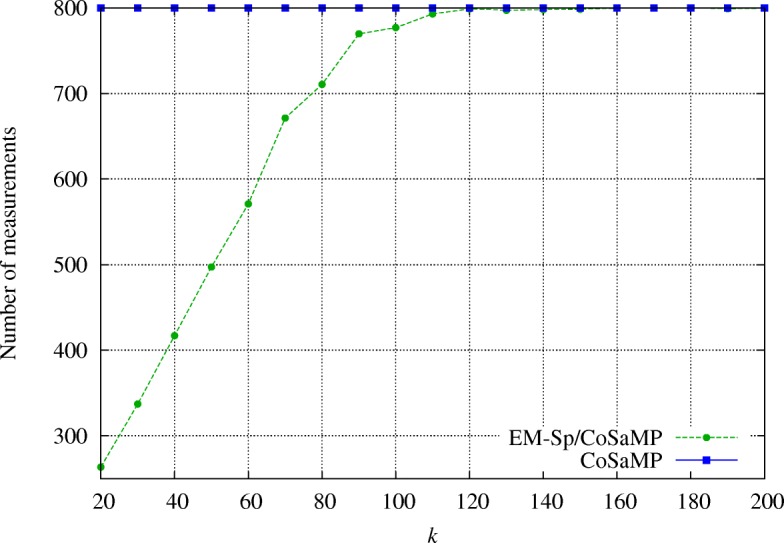
Recovery experiment with unknown *k*: EM-Sp/CoSaMP saves a significant number of measurements with respect to pure CoSaMP

**Fig. 8:**
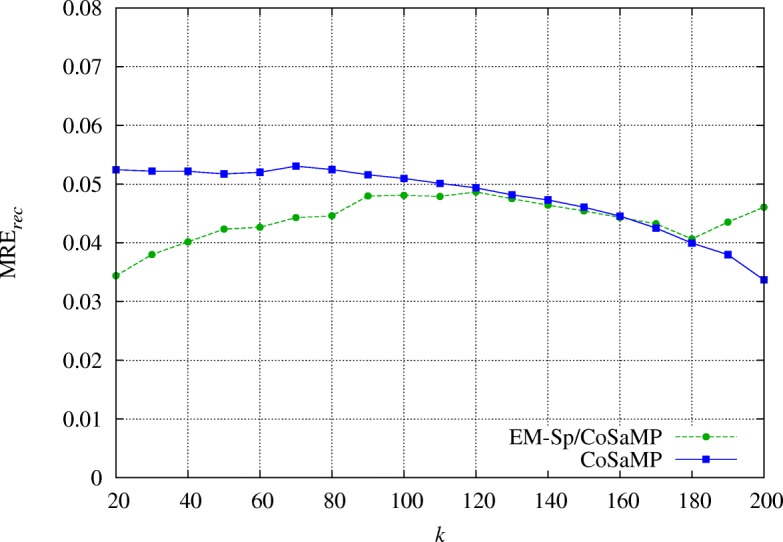
The gain obtained by EM-Sp/CoSaMP in terms of measurements has no price in terms of accuracy: the recovery mean relative error is close to that of pure CoSaMP

We show the results averaged over 100 random experiments. In Fig. 7, we compare the number of measurements used for recovery, as a function of the sparsity degree *k*: a substantial gain is obtained in terms of measurements by EM-Sp/CoSaMP, with no significant accuracy loss. In Fig. 8, we can see that CoSaMP and EM-Sp/CoSaMP algorithms achieve similar MRE _rec_.

## Conclusions

In this paper, we have proposed an iterative algorithm for the estimation of the signal sparsity starting from compressive and noisy projections obtained via sparse random matrices. As a first theoretical contribution, we have demonstrated that the estimator is consistent in the noise-free setting and we characterized its asymptotic behavior for different regimes of the involved parameters, namely the sparsity degree *k*, the number of measurements *m*, and the sensing matrix sparsity parameter *γ*. Then, we have showed that in the noisy setting, the projections can be approximated using a 2-GMM, for which the EM algorithm provides an asymptotically optimal estimator.

Numerical results confirm that the 2-GMM approach is effective for different signal models and outperforms methods known in the literature, with no substantial increase of complexity. The proposed algorithm can represent a useful tool in several applications, including the estimation of signal sparsity before reconstruction in a sequential acquisition framework, or the estimation of support overlap between correlated signals. An important property of the proposed method is that it does not rely on the knowledge of the actual sensing matrix, but only on its sparsity parameter *γ*. This enables applications in which one is not interested in signal reconstruction, but only in embedding the sparsity degree of the underlying signal in a more compact representation.

## Appendix

### Proofs of results in Section 4

#### Proof of Proposition 1

It should be noticed that $ \mathbb {P}(\omega ^{\star }_{i}=0)=(1-\gamma)^{\theta n}$, where *θ*∈[ 0,1] is the parameter to be optimized. Since the rows of the matrix *A* are independent, 5 so are $\omega _{i}^{\star }$, and considering that the event $\omega ^{\star }_{i}=0$ is equivalent to the event that the support of *i*th row of *A* is orthogonal to the support of signal *x*, the ML estimation computes

43 $$\begin{array}{*{20}l} {}\widehat{\theta}_{o}&=\underset{{\theta\in[0,1]}}{\mathrm{argmax\,}}\log f(\omega^{\star}|\theta)=\sum_{i=1}^{m}f(\omega^{\star}_{i}|\theta) \end{array} $$

44 $$\begin{array}{*{20}l} &=\!\underset{{\theta\in[0,1]}}{\mathrm{argmax\,}}\sum_{i=1}^{m}\log\left[ \left(1\,-\,(1-\gamma)^{\theta n}\right)^{\omega^{\star}_{i}}(1-\gamma)^{{\theta n}\left(1-\omega^{\star}_{i}\right)}\right] \end{array} $$

from which

45 $$ \widehat{\theta}_{o}=\frac{\log\left(1-\frac{\|\omega^{\star}\|_{0}}{m}\right)}{n\log(1-\gamma)}.  $$

We conclude that $\widehat {k}_{o}=\widehat {\theta }_{o}n$. □

#### Proof of Theorem 2

Let us consider $\omega ^{\star }_{i}$ as defined in (15) and let $\widehat {p}_{k}=\frac {\|\omega ^{\star }\|_{0}}{m}$ where the index emphasizes the dependence on the sparsity degree. We thus have:

46 $$ {\begin{aligned} \mathbb{P}\left(e(\widehat{k}_{o},k)>\epsilon\right)&=\mathbb{P}\left(\frac{\left|\widehat{k}_{o}-k\right|}{k}>\epsilon\right)\\ &=\mathbb{P}\left(\frac{\left|\log\left(1-\widehat{p}_{k}\right)-\log(1-p_{k})\right|}{\left|k\log(1-\gamma)\right|}>\epsilon\right). \end{aligned}}  $$

Since |*k* log(1−*γ*)|=|log(1−*γ*)^*k*^|=− log(1−*p*_*k*_), we obtain

47 $$\begin{array}{*{20}l} {}\mathbb{P}\left(e\left(\widehat{k}_{o},k\right)>\epsilon\right)&=\mathbb{P}\left({\left|\log\left(\frac{1-\widehat{p}_{k}}{1-p_{k}}\right)\right|}>-\epsilon\log (1-p_{k})\right) \end{array} $$

48 $$\begin{array}{*{20}l} &=\mathbb{P}\left({\left|\log\left(1+\frac{p_{k}-\widehat{p}_{k}}{1-p_{k}}\right)\right|}>-\epsilon\log (1-p_{k})\right) \end{array} $$

49 $$\begin{array}{*{20}l} &=\mathbb{P}\left({\log\left(1+\frac{p_{k}-\widehat{p}_{k}}{1-p_{k}}\right)}>-\epsilon\log (1-p_{k})\right) \end{array} $$

50 $$\begin{array}{*{20}l} &\quad+\mathbb{P}\left({\log\left(1+\frac{p_{k}-\widehat{p}_{k}}{1-p_{k}}\right)}<\epsilon\log (1-p_{k})\right) \end{array} $$

51 $$\begin{array}{*{20}l} &=\mathbb{P}\left({p_{k}-\widehat{p}_{k}}>\left(\mathrm{e}^{-\epsilon\log (1-p_{k})}-1\right)(1-p_{k})\right) \end{array} $$

52 $$\begin{array}{*{20}l} &\quad+\mathbb{P}\left({p_{k}-\widehat{p}_{k}}<\left(\mathrm{e}^{\epsilon\log (1-p_{k})}-1\right)(1-p_{k})\right) \end{array} $$

53 $$\begin{array}{*{20}l} &\quad\leq\mathbb{P}\left(\left|{p_{k}-\widehat{p}_{k}}\right|>\xi_{k}\right) \end{array} $$

with

54 $$\begin{array}{*{20}l} {}\xi_{k}&\,=\,{(1\,-\,p_{k})}\min\left\{\left(\mathrm{e}^{-\epsilon\log (1-p_{k})}-1\right),\left(1-\mathrm{e}^{\epsilon\log (1-p_{k})}\right)\right\} \end{array} $$

55 $$\begin{array}{*{20}l} &\!={(1-p_{k})}\left(1-\mathrm{e}^{\epsilon\log (1-p_{k})}\right). \end{array} $$

It should be noticed that $p_{k}=\mathbb {E}\left [\widehat {p}_{k}\right ]$, hence applying the Chernoff-Hoeffding theorem [38], the above tail probability is upper bounded as

56 $$\begin{array}{*{20}l} \mathbb{P}\left(e\left(\widehat{k}_{o},k\right)>\epsilon\right)\leq 2\mathrm{e}^{-2m\xi_{k}^{2}} \end{array} $$

and we obtain the first part of the statement. Choosing

57 $$\epsilon=\frac{\log\left(1-\frac{\sqrt{\rho}}{1-p_{k}}\sqrt{\frac{\log m}{m}}\right)}{\log (1-p_{k})} $$

for some *ρ*>1/2, we get

58 $$\begin{array}{*{20}l} \mathbb{P}\left(e\left(\widehat{k}_{o},k\right)>\epsilon\right)&\leq 2\mathrm{e}^{-2m\left(1-p_{k}\right)^{2}\left(1-\mathrm{e}^{\epsilon\log\left(1-p_{k}\right)}\right)^{2}} \end{array} $$

59 $$\begin{array}{*{20}l} &=2\mathrm{e}^{-2m\left(1-p_{k}\right)^{2}\left(1-\mathrm{e}^{{\log\left(1\,-\,\frac{\sqrt{\rho}}{1-p_{k}}\sqrt{\frac{\log m}{m}}\right)}}\right)^{2}} \end{array} $$

60 $$\begin{array}{*{20}l} &=2\mathrm{e}^{-2\rho \log m}=\frac{2}{m^{2\rho}} \end{array} $$

and from Borel-Cantelli Lemma [39], we conclude that

$$\mathbb{P}\left(\limsup_{k\rightarrow\infty}\left\{e\left(\widehat{k}_{o},k\right)\geq\epsilon_{k}\right\}\right)=0. $$

□

#### Proof of Theorem 3

From Theorem 2, we have

$$\mathbb{P}\left(\left\{e\left(\widehat{k}_{o},k\right)\geq\epsilon_{k}\right\}\right)\leq\frac{2}{m^{2\rho}},$$

and combining the hypothesis *m*/ log(*m*)≥*g*(*k*), we get

$$\mathbb{P}\left(\left\{e\left(\widehat{k}_{o},k\right)\geq\epsilon_{k}\right\}\right)\leq \frac{2}{(g(k))^{2\rho}(\log m)^{2\rho}}\leq \frac{1}{(g(k))^{2\rho}} $$

where the last inequality is obtained noticing that log*m*≥2 definitely as *m*→*∞*. We distinguish now the different cases

- If *ψ*(*k*)→*∞* as *k*→*∞*, then the function *g* is defined as *g*(*k*)=*Ω*(e^2*ψ*(*k*)^) from which we get that also *g*(*k*)→*∞*
- If *ψ*(*k*)=*Θ*(1) as *k*→*∞*, then the function *g* is defined as *g*(*k*)→*∞* for *k*→*∞* from which we get that also *g*(*k*)→*∞*;
- If *ψ*(*k*)=*o*(1) as *k*→*∞*, then the function *g* is defined as *g*(*k*)=*Ω*(*ψ*(*k*)^−2(1+*ε*)^), for any *ε*>0 from which we get that also *g*(*k*)→*∞*

Since in all cases (a), (b), and (c), the function *g*(*k*)→*∞*, we conclude $\mathbb {P}\left (\left \{e\left (\widehat {k}_{o},k\right)\geq \epsilon _{k}\right \}\right)\longrightarrow 0$ and the Eq. (22) can be deduced.

We now prove that *ε*_*k*_ tends to zero as *k*→*∞*. Notice that being *ψ*(*k*)=*o*(*k*) as *k*→*∞*

61 $$\begin{array}{*{20}l} {}p_{k}:&=1-(1-\gamma)^{k}=1-\left(1-\frac{\psi(k)}{k}\right)^{k}\sim 1-\mathrm{e}^{-\psi(k)} \end{array} $$

as *k*→*∞*. We have

62 $$\begin{array}{*{20}l} \epsilon_{k}&=\frac{\log\left(1-\frac{\sqrt{\rho}}{1-p_{k}}\sqrt{\frac{\log m}{m}}\right)}{\log (1-p_{k})} \end{array} $$

63 $$\begin{array}{*{20}l} &\sim\frac{\log\left(1-{\sqrt{\rho}\mathrm{e}^{\psi(k)}}\sqrt{\frac{\log m}{m}}\right)}{-{\psi(k)}}. \end{array} $$

We have

- If *ψ*(*k*)→*∞* then *ε*_*k*_=*O*(*ψ*(*k*)^−1^);
- If *ψ*(*k*)=*Θ*(1) then *ε*_*k*_=*O*(*g*(*k*)^−1/2^);
- If *ψ*(*k*)→0 then *ε*_*k*_=*O*(*ψ*(*k*)^*ε*^).

We conclude that in all three cases (a), (b), and (c) the threshold $ \epsilon _{k}\stackrel {k\rightarrow \infty }{\longrightarrow }0. $

#### Proof of Theorem 4

Let *ε*_*k*_ be defined as in (23). From Lemma 2, we have, for some *ρ*>1/2,

64 $$\begin{array}{*{20}l} \mathbb{P}\left(\left\{e\left(\widehat{k}_{o},k\right)\geq\epsilon_{k}\right\}\right)\leq\frac{2}{m^{2\rho}}\leq \frac{1}{k^{2\rho}} \end{array} $$

where the last inequality is obtained noticing that

65 $$m\geq \max\left\{k,\min\left\{\ell\in\mathbb{N}:\frac{\ell}{\log \ell}\geq g(k)\right\}\right\}\geq k $$

definitely. Since 2*ρ*>1, from the Borel-Cantelli lemma, we deduce that

66 $$ \mathbb{P}\left(\limsup_{k\rightarrow\infty}\left\{e\left(\widehat{k}_{o},k\right)\geq\epsilon_{k}\right\}\right)=0.  $$

Being log(*m*)/*m*≥*g*(*k*), then *ε*_*k*_→0 as *k*→*∞*, and we conclude that

67 $$ \mathbb{P}\left(\limsup_{k\rightarrow\infty}e\left(\widehat{k}_{o},k\right)=0\right)=1.  $$

### Proof of Theorem 5

In this section, we prove Theorem 5.

#### **Lemma 1**

Let *A* be chosen from the *γ*-sparsified Gaussian ensemble uniformly at random and *y* be given in (14), *p*_*k*_=1−(1−*γ*)^*k*^, and  then

$${\begin{aligned} \mathbb{E}\left[\text{Var}(y_{i})|\omega_{i}=1\right]&=\frac{\|x\|^{2}}{p_{k}}+\sigma^{2}\\ \text{Var}\left[\text{Var}(y_{i})|\omega_{i}=1\right]&=\frac{1}{p_{k}}\left(\frac{\sum_{\ell=1}^{n}x_{\ell}^{4}}{\gamma}+2\sum_{\ell>j}x_{\ell}^{2}x_{j}^{2}-\frac{\|x\|^{4}}{p_{k}}\right) \end{aligned}} $$

#### *Proof*

We recall $y_{i}=\sum _{j=1}^{n}A_{ij}x_{j}+\eta _{i}$ with *η*_*i*_∼N(0,*σ*^2^). As already noticed throughout the paper, the measurement *y*_*i*_ is a mixture of Gaussians with zero mean and variance depending on the overlap between the support of the *i*th row of *A* and supp(*x*). Suppose that *S*⊆supp(*x*) is this overlap which happens with probability *p*_*S*_=(1−*γ*)^*k*−|*S*|^*γ*^|*S*|^, then the variance of the Gaussian is given by $\alpha _{S}=\frac {\|x_{S}\|^{2}}{\gamma }+\sigma ^{2}$. Standard computations lead to

68 $$\begin{array}{*{20}l} {}\mathbb{E}\left[\text{Var}(y_{i})|\omega_{i}=1\right]&=\sum_{S\subseteq\text{supp}(x):S\neq\emptyset}\frac{p_{S}}{p_{k}}\left(\frac{\|x_{S}\|^{2}}{\gamma}+\sigma^{2}\right) \end{array} $$

69 $$\begin{array}{*{20}l} &=\sum_{S\subseteq\text{supp}(x):S\neq\emptyset}\frac{(1-\gamma)^{k-|S|}\gamma^{|S|}}{p_{k}}\left(\frac{\|x_{S}\|^{2}}{\gamma}\right)+\sigma^{2} \end{array} $$

70 $$\begin{array}{*{20}l} &=\frac{1}{p_{k}}\sum_{S\subseteq\text{supp}(x):S\neq\emptyset}{(1-\gamma)^{k-|S|}\gamma^{|S|-1}}{\sum_{\ell\in S}x_{\ell}^{2}}+\sigma^{2}. \end{array} $$

We notice that, fixed a component *ℓ*∈supp(*x*), we have exactly ${k-1\choose s-1} $ possible sets of cardinality *s* containing *ℓ*, i.e., the number of selections of the remaining *s*−1 objects among *k*−1 positions. This observation and the fact *x*_*ℓ*_=0,∀*ℓ*∉supp(*x*) leads to

71 $$\begin{array}{*{20}l} {}\mathbb{E}\left[\text{Var}(y_{i})|\omega_{i}=1\right]&=\frac{1}{p_{k}}\sum_{\ell=1}^{n}\sum_{s=1}^{k}{(1-\gamma)^{k-s}\gamma^{s-1}}{k-1\choose s-1}x_{\ell}^{2}+\sigma^{2} \end{array} $$

72 $$\begin{array}{*{20}l} &=\frac{1}{p_{k}}\sum_{\ell=1}^{n}\sum_{s=0}^{k-1}{(1-\gamma)^{k-s-1}\gamma^{s}}{k-1\choose s}x_{\ell}^{2}+\sigma^{2} \end{array} $$

73 $$\begin{array}{*{20}l} &=\frac{1}{p_{k}}\sum_{\ell=1}^{n}x_{\ell}^{2}+\sigma^{2}. \end{array} $$

We compute now

74 $$\begin{array}{*{20}l} &{}\text{Var}\left[\text{Var}(y_{i})|\omega_{i}=1\right] \end{array} $$

75 $$\begin{array}{*{20}l} &=\sum_{S\subseteq\text{supp}(x)}\frac{p_{S}}{p_{k}}\left(\frac{\|x_{S}\|^{2}}{\gamma}+\sigma^{2}\right)^{2} -\left(\frac{\|x\|^{2}}{p_{k}}+\sigma^{2}\right)^{2} \end{array} $$

76 $$\begin{array}{*{20}l} &=\sum_{S\subseteq\text{supp}(x)}\frac{p_{S}}{p_{k}}\left(\frac{\|x_{S}\|^{4}}{\gamma^{2}}+\frac{2\|x_{S}\|^{2}\sigma^{2}}{\gamma}+\sigma^{4}\right) \end{array} $$

77 $$\begin{array}{*{20}l} &\quad-\left(\frac{\|x\|^{4}}{p_{k}^{2}}+2\frac{\|x\|^{2}}{p_{k}}\sigma^{2}+\sigma^{4}\right) \end{array} $$

78 $$\begin{array}{*{20}l} &=\sum_{S\subseteq\text{supp}(x)}\frac{p_{S}}{p_{k}}\frac{\|x_{S}\|^{4}}{\gamma^{2}}-\frac{\|x\|^{4}}{p_{k}^{2}} \end{array} $$

79 $$\begin{array}{*{20}l} &=\frac{1}{p_{k}}\left(\sum_{S\subseteq\text{supp}(x)}(1-\gamma)^{k-|S|}\gamma^{|S|}\frac{\|x_{S}\|^{4}}{\gamma^{2}}-\frac{\|x\|^{4}}{p_{k}}\right) \end{array} $$

80 $$\begin{array}{*{20}l} &=\sum_{S\subseteq\text{supp}(x)}\frac{(1-\gamma)^{k-|S|}\gamma^{|S|-2}}{p_{k}}{\sum_{\ell\in S}x_{\ell}^{4}} \end{array} $$

81 $$\begin{array}{*{20}l} &\quad+\sum_{S\subseteq\text{supp}(x)}\frac{(1-\gamma)^{k-|S|}\gamma^{|S|-2}}{p_{k}}\sum_{\ell\neq j\in S}x_{\ell}^{2}x_{j}^{2}-\frac{\|x\|^{4}}{p_{k}^{2}} \end{array} $$

As before we notice that, fixed the component *ℓ*, we have exactly ${k-1\choose s-1} $ possible sets of cardinality *s* containing *ℓ*. Analogously, the couple *ℓ*,*j* with *ℓ*≠*j* is contained in ${k-2\choose s-2}$ possible sets of cardinality *s*. We thus have

82 $$\begin{array}{*{20}l} &\text{Var}\left[\text{Var}(y_{i})|\omega_{i}=1\right] \end{array} $$

83 $$\begin{array}{*{20}l} &=\sum_{s=1}^{k}\frac{(1-\gamma)^{k-s}\gamma^{s-2}}{p_{k}}{k-1\choose s-1}\sum_{\ell=1}^{n}x_{\ell}^{4} \end{array} $$

84 $$\begin{array}{*{20}l} &\quad+\sum_{s=2}^{k}\frac{(1-\gamma)^{k-s}\gamma^{s-2}}{p_{k}}{k-2\choose s-2}\sum_{i\neq j}x_{\ell}^{2}x_{j}^{2} -\frac{\|x\|^{4}}{p_{k}^{2}}. \end{array} $$

We conclude

85 $$\begin{array}{*{20}l} &\text{Var}\left[\text{Var}(y_{i})|\omega_{i}=1\right] \end{array} $$

86 $$\begin{array}{*{20}l} &\quad=\frac{1}{p_{k}}\left(\frac{\sum_{\ell=1}^{n}x_{\ell}^{4}}{\gamma}+\sum_{i\neq j}x_{\ell}^{2}x_{j}^{2}-\frac{\|x\|^{4}}{p_{k}}\right) \end{array} $$

□

### Proof of Theorem 5

Let *ψ*(*k*)=*γk* and consider the sequence of probability density functions

$${\begin{aligned} f_{k}(\zeta)&=\sum_{S\subseteq\text{supp}(x)}p_{S}\phi\left(\zeta|\alpha_{S}\right)\\ f^{\textrm{2-GMM}}_{k}(\zeta)&=(1-p_{k})\phi\left(\zeta|\sigma^{2}\right)+p_{k}\phi\left(\zeta\Big|\sigma^{2}+\frac{\|x\|^{2}}{p_{k}}\right), \end{aligned}} $$

where $\alpha _{S}=\frac {\|x_{S}\|^{2}}{\gamma }+\sigma ^{2}$ and *p*_*S*_=(1−*γ*)^*k*−|*S*|^*γ*^|*S*|^. Let $\bar {\alpha }=\mathbb {E}\left [\text {Var}(y_{i})|\omega _{i}=1\right ]=\sigma ^{2}+\frac {\|x\|^{2}}{p_{k}}$ (see Lemma 1) and denote $\mathcal {S}$ the set of possible subsets of supp(*x*), we thus have

87 $$ \begin{aligned} &\left\|f_{k}-f^{\textrm{2-GMM}}_{k}\right\|_{\mathrm{K}}\\ &\ =\sup_{t\in\mathbb{R}}\left|\sum_{S\in\mathcal{S}\setminus \{\emptyset\}}{p_{S}}\int_{-\infty}^{t}\phi\left(\zeta|\alpha_{S}\right)-p_{k}\int_{-\infty}^{t} \phi\left(\zeta|\bar{\alpha}\right)\right|\\ &\ =\frac{p_{k}}{2}\sup_{t\in\mathbb{R}}\left|\sum_{S\in\mathcal{S}\setminus \{\emptyset\}}\frac{p_{S}}{p_{k}}\text{erf}\left(\frac{t}{\sqrt{2\alpha_{S}}}\right)-\text{erf}\left(\frac{t}{\sqrt{2\bar{\alpha}}}\right)\right| \end{aligned}  $$

where erf is the Gauss error function. Let $g:\mathbb {R}^{2}\rightarrow \mathbb {R}$ be the function

88 $$ g(t,\alpha):=\text{erf}\left(\frac{t}{\sqrt{2\alpha}}\right)  $$

and by the Lagrange Theorem [40], we obtain

89 $$ g(t,\alpha)=g(t,\bar{\alpha})+\frac{\partial g}{\partial{\alpha}}(t,\bar{\alpha})(\alpha-\bar{\alpha})+\frac{1}{2}\frac{\partial^{2} g}{\partial{\alpha}^{2}}(t,\xi)(\alpha-\bar{\alpha})^{2}  $$

with $\xi (\alpha)\in (\min \{\bar {\alpha },\alpha \},\max \{\alpha,\bar {\alpha }\})$. It should be noticed that the first-order term in Taylor’s expansion of *g*(*t*;*α*) vanishes due to conditional mean result from Theorem 2

90 $$\begin{array}{*{20}l} &\sum_{S\in\mathcal{S}\setminus \{\emptyset\}}\frac{p_{S}}{p_{k}}g(t,\alpha_{S}) \end{array} $$

91 $$\begin{array}{*{20}l} &\quad =g(t,\bar{\alpha})+\frac{1}{2}\sum_{S\in\mathcal{S}\setminus \{\emptyset\}}\frac{p_{S}}{p_{k}}\left[\frac{\partial^{2} g}{\partial{\alpha}^{2}}(t,\xi(\alpha_{S}))(\alpha_{S}-\bar{\alpha})^{2}\right] \end{array} $$

with $\xi (\alpha _{S})\in (\min \{\bar {\alpha },\alpha \},\max \{\alpha,\bar {\alpha }\})\subseteq (\lambda ^{2}/\gamma,\|x\|^{2}/\gamma)$. Putting this expression into (87), we obtain

92 $$\begin{array}{*{20}l} &\left\|f_{k}-f^{\textrm{2-GMM}}_{k}\right\|_{\mathrm{K}} \end{array} $$

93 $$\begin{array}{*{20}l} &\leq\quad \left|\frac{p_{k}}{4}\sum_{S\in\mathcal{S}\setminus \{\emptyset\}}\frac{p_{S}}{p_{k}}\left[\frac{\partial^{2} g}{\partial{\alpha}^{2}}(t,\xi(\alpha_{S}))(\alpha_{S}-\bar{\alpha})^{2}\right]\right| \end{array} $$

94 $$\begin{array}{*{20}l} &\leq\quad \frac{p_{k}}{4}\sum_{S\in\mathcal{S}\setminus \{\emptyset\}}\frac{p_{S}}{p_{k}}\left|\frac{\partial^{2} g}{\partial{\alpha}^{2}}(t,\xi(\alpha_{S}))\right|(\alpha_{S}-\bar{\alpha})^{2} \end{array} $$

95 $$\begin{array}{*{20}l} &\leq\quad \frac{p_{k}}{4}G_{\max}\sum_{S\in\mathcal{S}\setminus \{\emptyset\}}\frac{p_{S}}{p_{k}}(\alpha_{S}-\bar{\alpha})^{2} \end{array} $$

where

96 $$G_{\max}=\max_{\xi\in[\lambda^{2}/\gamma,\|x\|^{2}/\gamma]}\sup_{t\in\mathbb{R}}\left|\frac{\partial^{2} g}{\partial{\alpha}^{2}}(t,\xi)\right|  $$

and

97 $$ \frac{\partial^{2} g}{\partial{\alpha}^{2}}(t,\xi)=\frac{1}{2\sqrt{2\pi}}\mathrm{e}^{\frac{-t^{2}}{2\xi}}\frac{t}{\xi^{5/2}}\left(3-\frac{t^{2}}{\xi}\right)  $$

Through standard computations, we see that the maximizing value is obtained for $t=\sqrt {(3-\sqrt {6})\xi }$

98 $$\begin{array}{*{20}l} G_{\max}&=\max_{\xi\in[\lambda^{2}/\gamma,\|x\|^{2}/\gamma]}\sup_{t\in\mathbb{R}}\left|\frac{1}{2\sqrt{2\pi}}\mathrm{e}^{\frac{-t^{2}}{2\xi}}\frac{t}{\xi^{5/2}}\left(3-\frac{t^{2}}{\xi}\right)\right| \end{array} $$

99 $$\begin{array}{*{20}l} &=C\max_{\xi\in[\lambda^{2}/\gamma,\|x\|^{2}/\gamma]}\xi^{-2}=C\frac{\gamma^{2}}{\lambda^{4}}. \end{array} $$

with

100 $$ C=\frac{\sqrt{\left(3-\sqrt{6}\right)}\sqrt{3}}{2\sqrt{\pi}}\mathrm{e}^{\frac{-\left(3-\sqrt{6}\right)}{2}}.  $$

Finally, considering

101 $$\begin{array}{*{20}l}\sum_{S\in\mathcal{S}\setminus \{\emptyset\}}\frac{p_{S}}{p_{k}}(\alpha_{S}-\bar{\alpha})^{2}=\text{Var}\left[\text{Var}(y_{i})|\omega_{i}=1\right] \end{array} $$

and using Lemma 1, we conclude

102 $$\begin{array}{*{20}l} &\left\|f_{k}-f^{\textrm{2-GMM}}_{k}\right\|_{\mathrm{K}} \end{array} $$

103 $$\begin{array}{*{20}l} &\quad\leq\frac{C^{\prime}\gamma^{2}}{\lambda^{4}}\left(\frac{\sum_{i=1}^{n}x_{i}^{4}}{\gamma}+\sum_{i\neq j}x_{i}^{2}x_{j}^{2}-\frac{\|x\|^{4}}{p_{k}}\right) \end{array} $$

with *C*^′^=*C*/4.

#### Proof of Corollary 1

From Theorem 5, we have

104 $$\begin{array}{*{20}l} {}\left\|f_{k}-f^{\textrm{2-GMM}}_{k}\right\|_{\mathrm{K}}&\leq\frac{C\gamma^{2}}{\lambda^{4}}\left(\frac{\sum_{i=1}^{n}x_{i}^{4}}{\gamma}+\sum_{i\neq j}x_{i}^{2}x_{j}^{2}-\frac{\|x\|^{4}}{p_{k}}\right) \end{array} $$

105 $$\begin{array}{*{20}l} &\leq C\left(\frac{\lambda_{\max}}{\lambda_{\min}}\right)^{4}(\psi(k)+\psi(k)^{2}). \end{array} $$

where *λ*_min_= min*i*|*x*_*i*_| and $\lambda _{\max }=\max _{\{i:\ x_{i}\neq 0\}}|x_{i}|$. The assertion is proved with *C*^′^=*C*(*λ*_max_/*λ*_min_)^4^.

## Abbreviations

2-GMM: Two-component Gaussian mixture model
CS: Compressed sensing
CoSaMP: Compressive sampling matching pursuit
DCT: Discrete cosine transform
EM: Expectation-maximization
ML: Maximum likelihood
MRE: Mean relative error
OMP: Orthogonal matching pursuit
SNR: Signal-to-noise ratio

## Footnotes

1 The code to reproduce the simulations proposed in this section is available at https://github.com/sophie27/sparsityestimation
